# Artificial intelligence in cardiovascular procedures: a bibliometric and visual analysis study

**DOI:** 10.1097/MS9.0000000000003112

**Published:** 2025-02-28

**Authors:** Koushik Rao Gadhachanda, Mohammed Dheyaa Marsool Marsool, Ali Bozorgi, Daniyal Ameen, Sandeep Samethadka Nayak, Amir Nasrollahizadeh, Abdulhadi Alotaibi, Alireza Farzaei, Mohammad-Hossein Keivanlou, Soheil Hassanipour, Ehsan Amini-Salehi, Anil Kumar Jonnalagadda

**Affiliations:** a Boston University Biology, Boston, Massachusetts, USA; b Department of Internal Medicine, Al-Kindy College of Medicine, University of Baghdad, Baghdad, Iraq; c Tehran Heart Center, Tehran University of Medical Sciences, Tehran, Iran; d Department of Internal Medicine, Yale New Haven Health Bridgeport Hospital, Bridgeport, Connecticut, USA; e Department of Medicine, Vision Colleges, Riyadh, Saudi Arabia; f Shahid Beheshti University of Medical Sciences, Tehran, Iran; g Guilan University of Medical Sciences, Rasht, Iran; h Department of Cardiology, John Peter Smith Hospital, Fort Worth, Texas

**Keywords:** artificial intelligence, cardiovascular surgery, machine learning

## Abstract

**Background:**

The integration of artificial intelligence (AI) into cardiovascular procedures has significantly advanced diagnostic accuracy, outcome prediction, and robotic-assisted surgeries. However, a comprehensive bibliometric analysis of AI’s impact in this field is lacking. This study examines research trends, key contributors, and emerging themes in AI-driven cardiovascular interventions.

**Methods:**

We retrieved relevant publications from the Web of Science Core Collection and analyzed them using VOSviewer, CiteSpace, and Biblioshiny to map research trends and collaborations.

**Results:**

AI-related cardiovascular research has grown substantially from 1993 to 2024, with a sharp increase from 2020 to 2023, peaking at 93 publications in 2023. The USA (127 papers), China (79), and England (31) were the top contributors, with Harvard University leading institutional output (17 papers). *Frontiers in Cardiovascular Medicine* was the most prolific journal. Core research themes included “machine learning,” “mortality,” and “cardiac surgery,” with emerging trends in “association,” “implantation,” and “aortic stenosis,” underscoring AI’s expanding role in predictive modeling and surgical outcomes.

**Conclusion:**

AI demonstrates transformative potential in cardiovascular procedures, particularly in diagnostic imaging, predictive modeling, and patient management. This bibliometric analysis highlights the growing interest in AI applications and provides a framework for integrating AI into clinical workflows to enhance diagnostic accuracy, treatment strategies, and patient outcomes.

## Introduction

In the 20th century, cardiac and cardiovascular procedures made remarkable progress despite facing initial challenges and some reluctance from the medical community^[1,2]^. This field expanded to new techniques such as minimally invasive surgeries, endoscopic and endovascular procedures, and even robotic surgeries that have become vital for treating a range of heart issues, including blocked arteries, valve problems, and congenital heart defects^[3-7]^. These recent advancements not only alleviated symptoms but also extended life expectancy, and enhanced patients’ overall quality of life^[8-11]^.

**HIGHLIGHTS**

- AI integration in cardiovascular procedures is advancing quickly; a bibliometric study is crucial to map research and guide future directions.
- Research surged post-2017, peaking in 2023, led by the U.S. Key themes included machine learning, mortality, and risk management, highlighting AI’s role in predictive modeling and surgical outcomes.
- AI shows transformative potential for cardiovascular surgery, requiring ongoing research and collaboration to optimize integration and patient’s care.

Artificial intelligence (AI) which includes machine learning (ML) and deep neural networks that are useful for interpreting complex medical images beyond human capacity, is widely used in modern society^[12,13]^. It helps us with medical applications, such as identifying pathological specimens and diagnosing conditions with remarkable accuracy^[14-17]^. AI enhances cardiovascular procedures by facilitating access to surgical data, improving diagnostic accuracy, and utilizing ML algorithms to predict outcomes. Additionally, robotic-assisted surgeries have emerged as a valuable complement to conventional methods^[18-21]^.

The objective of this study is to conduct a bibliometric analysis which is a method to evaluate scientific research and gain important insights into productivity and quality within scientific fields^[22]^, to examine the evolution and status of AI and cardiovascular surgeries. A bibliographic study is essential for this study to evaluate the rapidly growing AI literature in cardiovascular procedures, identifying key trends, significant contributions, and knowledge gaps, thus guiding future research, and informing clinical practices. Specifically, we aim to identify key trends, influential publications, prolific authors and institutions, and emerging research themes.

## Methods

### Data collection

**Figure 1.**
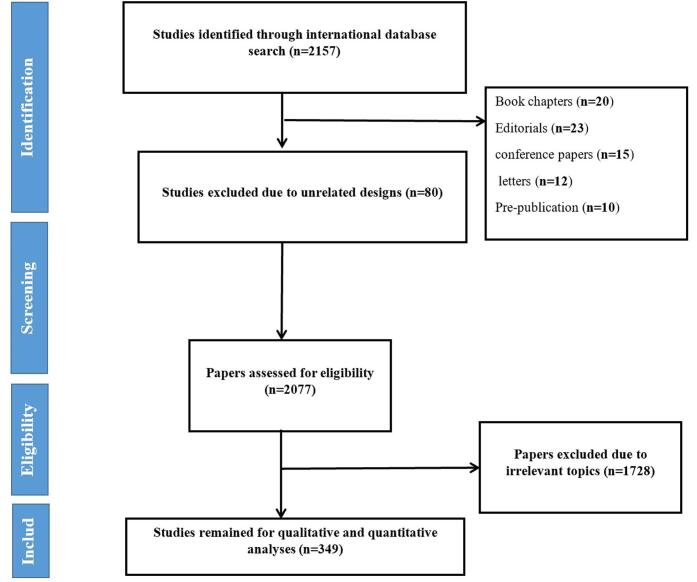
Study selection process.

We obtained data about published articles by carrying out a search of our main database, the Web of Science Core Collection, on 13 July 2024. This database, which includes more than 12 000 reliable articles, is well-known for its vast and reliable collection of information^[23-25]^. As shown in Supplementry Digital Content, Table S1 (http://links.lww.com/MS9/A771), we created a search strategy that employed multiple keywords to optimize the efficacy of our search. At first, we found 2157 objects. After that, we narrowed down our selection to 349 papers by eliminating studies unrelated to our research goals, book chapters, editorials, conference papers, letters, and pre-publication papers (Fig. 1).

### Data analysis

We used VOS viewer, CiteSpace, and Biblioshiny (R Bibliometrix package) due to their complementary strengths in visualizing and analyzing research trends to evaluate the relevant documents we downloaded from the Web of Science Core Collection. The data were then converted into plain text and CSV formats.

A potent tool for scientometric network analysis is VOSviewer, which was created by the Center for Science and Technology Studies at Leiden University in the Netherlands. It is especially proficient at creating maps from network data and offering visual representations of the connections found in scholarly literature. Network diagrams illustrating co-citation, co-occurrence, citation, and bibliographic coupling among publications, journals, authors, research institutes, nations, and keywords can be created with VOS viewer^[26-28]^.

Another essential instrument for our investigation was CiteSpace. CiteSpace is a Java application that integrates scientometrics and data visualization concepts to analyze citation visualizations. This innovative method explains the dynamics, structure, and patterns of distribution of scientific knowledge by creating detailed knowledge maps through the application of complex algorithms for data mining and thorough information analysis. CiteSpace is a tool that helps researchers identify major developments, notable studies, and emerging fields of interest in academic communication by providing visual representations of citation networks^[29]^.

Biblioshiny is an intuitive graphical user interface web application for the Bibliometrix R software that facilitates bibliometric analysis. Biblioshiny allows us to conduct a thorough analysis. It can do several tasks, including network analysis, descriptive data analysis, and bibliometric network visualization^[30]^.

## Results

### Publication trend

**Figure 2.**
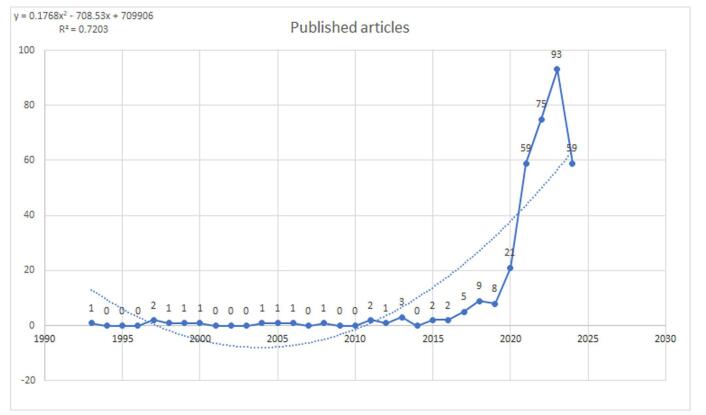
Trends in publication regarding artificial intelligence in cardiac procedures (dotted line shows the trend line).

A field’s research trends can be measured by the number of publications produced over a specific amount of time. Publications on AI in cardiovascular procedures were few between 1993 and 2016, indicating little effort in this field. Activity gradually increased between 2017 and 2019, suggesting an increasing interest in this field. There was notable growth from 2020 to 2024. There was a notable increase in scholarly activity and attention over this period, peaking in 2023 with the publication of 93 papers. This considerable rise highlights the growing attention being paid to the possible uses of AI in cardiovascular procedures. Nonetheless, 2024 saw a drop with only 59 publications, which was expected given the year ongoing (Fig. 2). These developments highlight the increasing awareness of the investigation into the novel possibilities of AI in the field of cardiovascular procedures.

**Figure 3.**
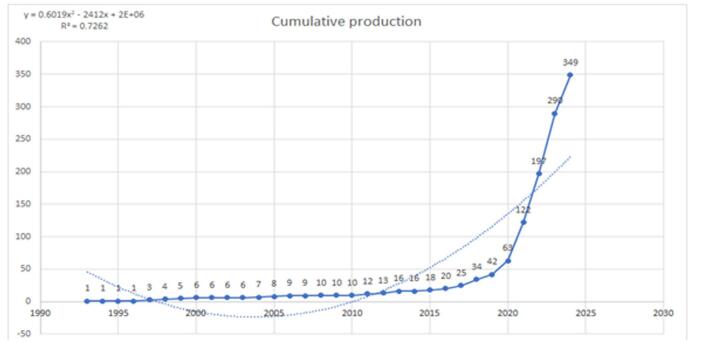
Cumulative publications regarding artificial intelligence in cardiac procedures (dotted line shows the trend line).

The cumulative production showed a significant increase over the period from 1993 to 2024. Initially, the growth was slow and steady, with cumulative values remaining below 10 until 2010. A slight increase is observed from 2011 to 2019, with values reaching 49 papers. From 2020 onward, the growth rate accelerated significantly. The cumulative production reached 122 in 2021 and then sharply increased to 349 in 2024 (Fig. 3).

### Countries and institutions

**Figure 4.**
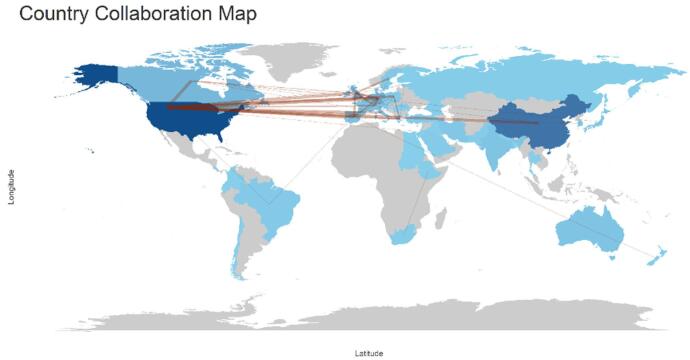
Countries collaboration in the field of artificial intelligence in cardiac procedures.

**Figure 5.**
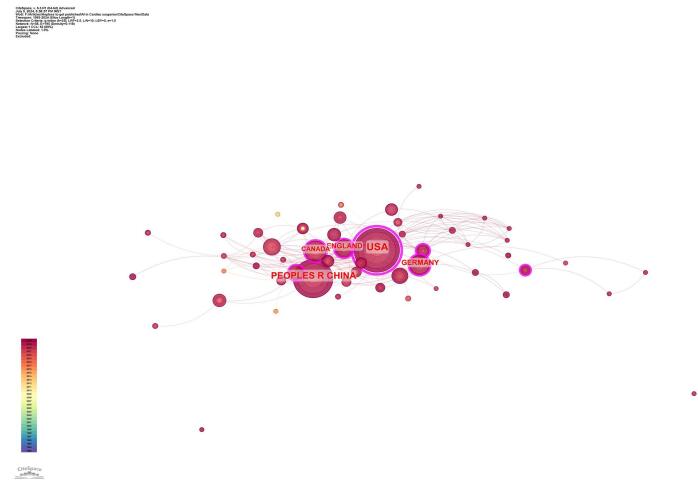
Countries with the high centrality in the field of artificial intelligence in cardiac procedures.

The examination of contributions by countries and institutions uncovers substantial engagement from several nations. Figure 4 shows the collaboration among countries. The analysis identified a total of 58 countries that have contributed to the field. Among these countries, 13 contributed to more than 10 papers. The United States had the highest number of papers (*n* = 127), followed by China (*n* = 79) and England (*n* = 31). Germany (*n* = 28), Canada (*n* = 22), the Netherlands (*n* = 16), Spain (*n* = 13), Italy (*n* = 13), France (*n* = 10), and Australia (*n* = 10) also made significant contributions. Table 1 displays the top 10 countries along with their number of publications and centrality values. The USA had the highest centrality (0.78), indicating its significant influence in the field, followed by Germany (0.16) and Spain (0.14). Figure 5 shows the countries with high centrality.

**Table 1:** Top 10 countries and institutions in the field of artificial intelligence in cardiac procedures.

Rank	Country	Contributions	Centrality	Institution	Contribution	Centrality
1	United States	127	0.78	Harvard University	17	0.18
2	China	79	0.06	Chinese Academy of Medical Science	16	0.08
3	England	31	0.11	Harvard Medical School	15	0.18
4	Germany	28	0.16	Mayo Clinic	15	0.01
5	Canada	22	0.12	Peking Union Medical College	15	0.07
6	Netherlands	16	0.08	University System of Ohio	11	0.10
7	Spain	13	0.14	Fu Wai Hospital—CAMS	11	0.00
8	Italy	13	0.00	German Centre for Cardiovascular Research	10	0.15
9	France	10	0.11	Cleveland Clinic Foundation	9	0.11
10	Australia	10	0.06	Johns Hopkins University	9	0.08

**Figure 6.**
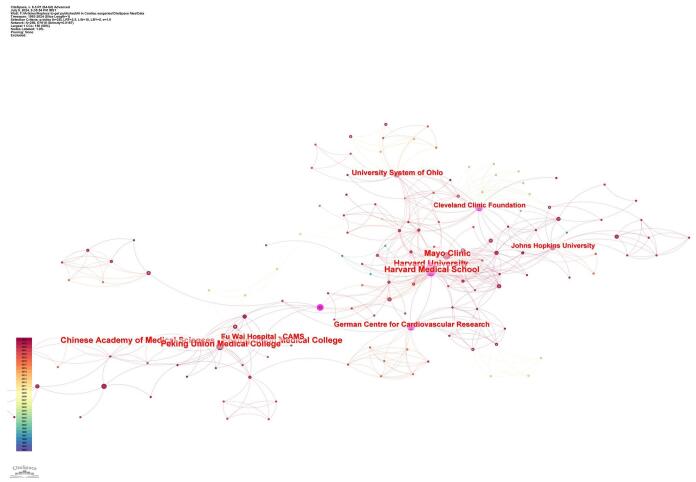
Institutions with the high centrality in the field of artificial intelligence in cardiac procedures.

A total of 781 institutions have worked together in this area overall. Harvard University (*n* = 17) was the institution with the most publications, closely followed by the Chinese Academy of Medical Science (*n* = 16). Peking Union Medical College and the Mayo Clinic each provided 15 papers. The University System of Ohio (*n* = 11), Fu Wai Hospital—CAMS (*n* = 11), German Centre for Cardiovascular Research (*n* = 10), Cleveland Clinic Foundation (*n* = 9), and Johns Hopkins University (*n* = 9) are among the other renowned organizations. Harvard University had high centrality values (0.18) followed by the German Centre for Cardiovascular Research (0.15) and Peking Union Medical College (0.15), indicating their prominent roles in this research domain (Table 1 and Fig. 6).

### Journals and co-cited journals

**Figure 7.**
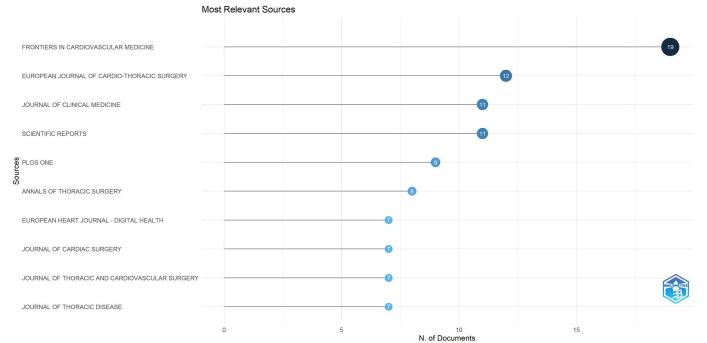
Top 10 journals in the field of artificial intelligence in cardiac procedures.

Our analysis identified 178 sources for publications in cardiovascular medicine and surgery. “Frontiers in Cardiovascular Medicine” had the highest number of documents (*n* = 19), followed by the “European Journal of Cardio-Thoracic Surgery” (*n* = 12), and both the “Journal of Clinical Medicine” and “Scientific Reports” (n =11 each). Other notable sources include “PLOS ONE” (*n* = 9), “Annals of Thoracic Surgery” (*n* = 8), and journals with seven documents each, such as “European Heart Journal—Digital Health,” “Journal of Cardiac Surgery,” “Journal of Thoracic and Cardiovascular Surgery,” and “Journal of Thoracic Disease” (Fig. 7).

**Figure 8.**
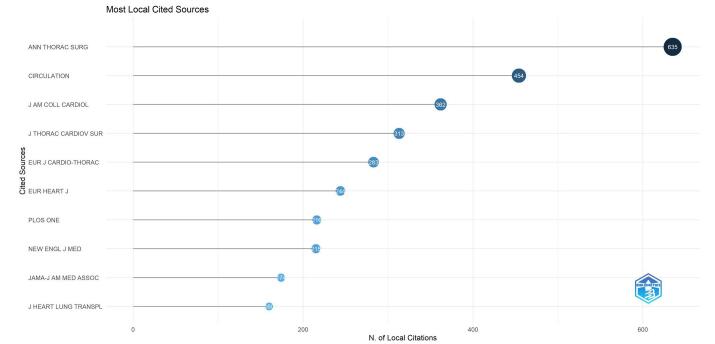
Top 10 co-cited journals in the field of artificial intelligence in cardiac procedures.

The analysis identified 2806 co-cited journals. Annals of Thoracic Surgery had the highest number of co-citations (*n* = 635), followed by “Circulation” (*n* = 454) and the “Journal of the American College of Cardiology” (*n* = 362). Other frequently co-cited journals include the “Journal of Thoracic and Cardiovascular Surgery” (*n* = 311), “European Journal of Cardio-Thoracic Surgery” (*n* = 283), and “European Heart Journal” (*n* = 244). Additional notable co-cited journals are “PLOS ONE” (*n* = 216), “New England Journal of Medicine” (*n* = 215), “Journal of the American Medical Association” (*n* = 174), and the “Journal of Heart and Lung Transplantation” (*n* = 160) (Fig. 8).

**Figure 9.**
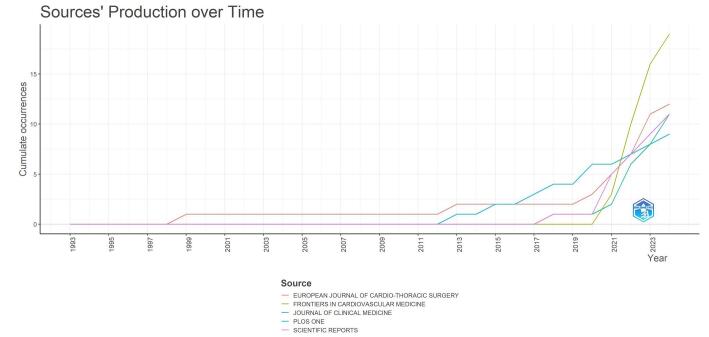
Journals’ productions over time in the field of artificial intelligence in cardiac procedures.

The analysis of publication production over time reveals trends in the research output of various journals. Figure 9 showcases the cumulative occurrences of publications from different sources, providing insights into the growth and development of the field. Between the late 1990s and 2011, there was a low level of research production in this field, with very few occurrences across multiple journals. During this period, both the *European Journal of Cardio-Thoracic Surgery and Scientific Reports* made contributions. From around 2012 onward, *PLOS ONE* also made contributions. The journal *Frontiers in Cardiovascular Medicine* faced a significant surge in articles, especially from the year 2020 forward. This journal is the most pronounced journal in this field regarding the number of publications. The overall trend indicates a sharp increase in the number of publications related to AI in cardiac imaging over the past decade, with a particularly notable rise from 2020 onward.

**Figure 10.**
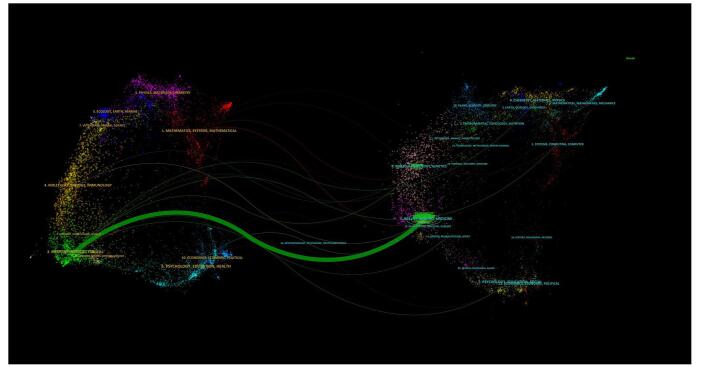
Dual overlay map of the citation patterns in the field of artificial intelligence in cardiac procedures.

Citation trends can be observed by the dual-map overlay illustrating journal linkages, where cited journals are on the right and citing journals appear on the left. The analysis indicated one main citation pattern (green line), which highlighted journals in medicine, medical, and clinical fields. These journals were frequently cited in *Health, Nursing, and Medicine* (Fig. 10).

### Cited and co-cited references

**Figure 11.**
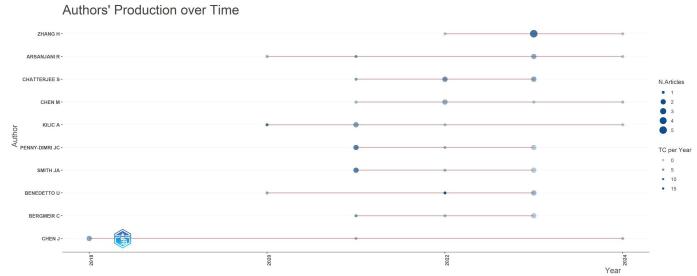
Authors production over time in the field of artificial intelligence in cardiac procedures.

A co-citation relationship is created between two references when they are mentioned simultaneously in the bibliography of another article. A total of 9999 co-cited references were recognized. The top 10 most often cited and co-cited articles within this discipline are highlighted in Table 2. The foundational literature and its influence on current research are showcased in this table, which offers insightful information on the most influential studies and their relationships. Figure 11 shows the authors production activity over time.

**Table 2:** Top 10 cited and co-cited references in the field of artificial intelligence in cardiac procedures.

Number	Title of most cited paper	Doi	Published year	Title of most co-cited paper	Doi	Published year
1	Tseng *et al*^[31]^	10.1186/s13054-020-03179-9	2020	Nashef *et al*^[32]^	10.1093/ejcts/ezs043	2012
2	Meyer *et al*^[33]^	10.1016/s2213-2600(18)30300-x	2018	Allyn *et al*^[34]^	10.1371/journal.pone.0169772	2017
3	Ortmaier^[35]^	10.1109/tbme.2005.855716	2005	Kilic *et al*^[36]^	10.1016/j.athoracsur.2019.09.049	2020
4	Shahian *et al*^[37]^	10.1016/j.athoracsur.2004.05.054	2004	Nashef *et al*^[38]^	10.1016/s1010-7940(99)00134-7	1999
5	Lee *et al*^[39]^	10.3390/jcm7100322	2018	Tseng *et al*^[31]^	10.1186/s13054-020-03179-9	2020
6	Allyn *et al*^[34]^	10.1371/journal.pone.0169772	2017	Mack *et al*^[40]^	10.1056/nejmoa1814052	2019
7	Nilsson *et al*^[41]^	10.1016/j.jtcvs.2005.12.055	2006	Lee *et al*^[39]^	10.3390/jcm7100322	2018
8	Tu *et al*^[42]^	10.1006/cbmr.1993.1015	1993	Lundenberg *et al*^[43]^	10.48550/arXiv.1705.07874	2017
9	Hernandez-Suarez *et al*^[42]^	10.1016/j.jcin.2019.06.013	2019	Deo *et al*^[44]^	10.1161/circulationaha.115.001593	2015
10	Lippmann *et al*^[45]^	10.1016/s0003-4975(97)00225-7	1997	Hernandez-Suarez *et al*^[42]^	10.1016/j.jcin.2019.06.013	2019

### Authors and co-cited authors

The analysis of authors contributing to the domain of AI in cardiac procedures revealed significant contributions from several researchers. Table 3 shows the top 10 authors based on the number of publications, citations, and co-citations within this specialized field.

**Table 3:** Top 10 Cited and co-cited authors in the field of artificial intelligence in cardiac procedures.

Number	Author with high number of publications	Number of publications	Author with high number of citations	Number of citations	The most co-cited authors	Number of citations
1	Reza Arsanjani	5	Sophie Provenchere	110	Arman Kilic	69
2	Arman Kilic	5	Johan Nilsson	83	Sam Nashef	67
3	Julian A. Smith	5	Mattias Ohlsson	80	SM O’Brien	42
4	Jahan C. Penny-Dimri	5	Arman Kilic	76	SM Lundberg	42
5	Umberto Benedetto	4	Umberto Benedetto	66		40
6	Arnaldo Dimagli	4	Arnaldo Dimagli	66	Umberto Benedetto	36
7	Shubhra Sinha	4	Shubhra Sinha	66	Leo Breiman	35
8	Joseph Schoepf	4	Gianni D. Angelini	50	Tian Qi Chen	33
9	Christopher Reid	4	Partho P. Sengupta	50	David Michael Shahian	32
10	Christoph Bergmeir	4	U. Joseph Schoepf	47	Jérôme Allyn	29

A total of 2502 authors were recognized for their contributions to the field; Reza Arsanjani, Arman Kilic, Julian A. Smith, and Jahan C. Penny-Dimri were in the lead with five publications each.

Sophie Provenchere was the leading author regarding citation impact with 110 citations; Johan Nilsson was the second author with 83 citations, and Mattias Ohlsson came in third with 80 citations.

Arman Kilic was the most co-cited author with 69 co-citations, followed by Sam Nashef with 67 co-citations. Sean M O’Brien and Scott M Lundberg had 42 co-citations each, and J Nilsson had 40 co-citations.

### Keyword trends, hotspots, cluster analysis

The analysis recognized 1378 keywords across the included studies. Table 4 shows the top 10 frequent keywords, recent keywords, and high central keywords.

**Table 4:** Top 10 frequent keywords, recent keywords, and high central keywords.

Number	Most occurred keywords	Most recent keywords	High central keywords	Centrality
1	Machine learning	170	Mortality	0.17
2	Mortality	87	Diagnosis	0.16
3	Cardiac surgery	66	Artificial intelligence	0.14
4	Artificial intelligence	58	Cardiac surgery	0.14
5	Risk	57	Society	0.13
6	Outcomes	52	Disease	0.13
7	Cardiac surgery	40	Morbidity	0.10
8	Prediction	36	Prediction	0.09
9	Score	32	Machine learning	0.09
10	Models	31	Score	0.07

**Figure 12.**
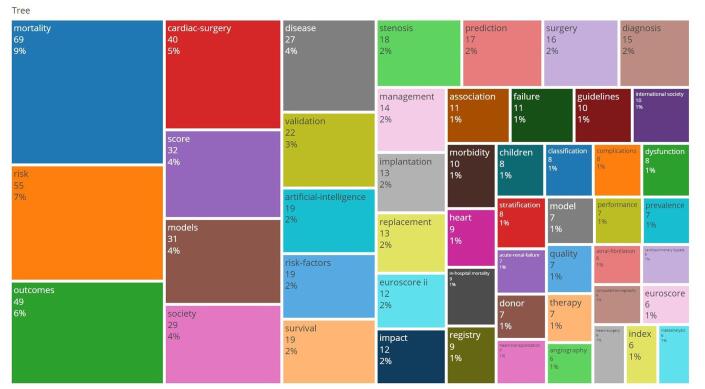
Word three map of the keywords in the field of artificial intelligence in cardiac procedures.

The most frequently occurring keywords were “machine learning” (*n* = 170), “mortality” (*n* = 87), “cardiac surgery” (*n* = 66), “artificial intelligence” (*n* = 58), and “risk” (*n* = 57). This high frequency indicates these are core areas of focus within the research on AI in cardiac procedures (Fig. 12). In cardiac procedures, the frequent use of the term “machine learning” underscores its pivotal role. For years, physicians have sought to identify, quantify, and interpret variable relationships to enhance patient care. AI and ML encompass a range of techniques that enable computers to perform these tasks by algorithmically learning efficient data representations.

When examining the centrality of keywords within the network, the top five most central keywords were “mortality” (0.17), “diagnosis” (0.16), “cardiac surgery” (0.14), “artificial intelligence” (0.14), and “validation” (0.14). These keywords indicate critical points of connection and influence within the research network. Patients undergoing cardiac surgery face high risks of complications, making preoperative risk-benefit evaluation crucial. Clinical risk scoring systems, though helpful, have limitations, especially for high-risk patients. AI may offer better predictive accuracy and validation by handling complex and nonlinear data relationships.

**Figure 13.**
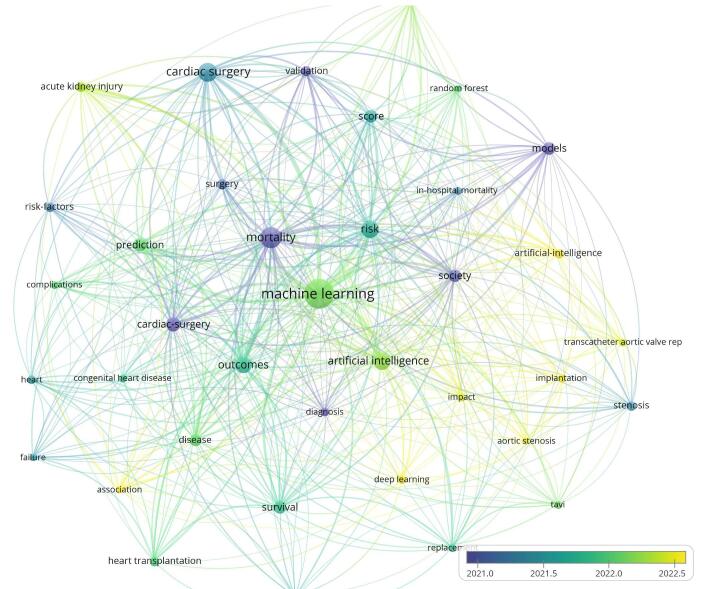
Overlay visualization keywords in the field of artificial intelligence in cardiac procedures.

**Figure 14.**
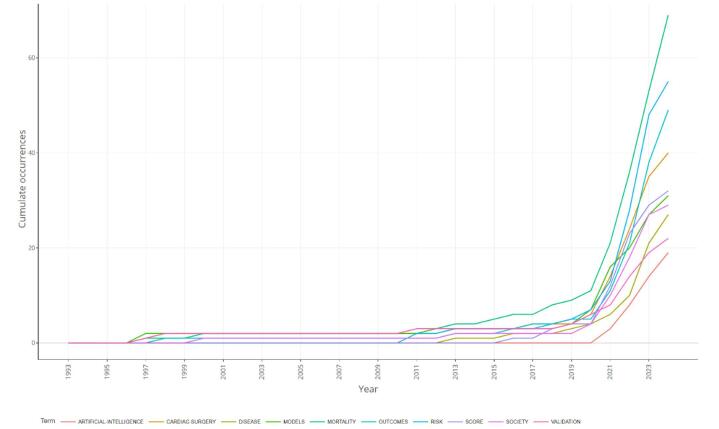
Frequency of keywords over time in the field of artificial intelligence in cardiac procedures.

The plot of the overlay visualization shows new trends and recent keywords. “Association,” “implantation,” “artificial intelligence,” “deep learning,” and “aortic stenosis” were the most recent keywords (Fig. 13). It may represent the role and efficacy of AI in predicting the aortic valve stenosis progression and the treatment outcome. Keywords like “mortality,” “outcomes,” and “risk” attracted a significant rise after 2010, showing a growing focus on the implications of AI in patient mortality and outcomes. This trend underscores the expanding role of AI in enhancing predictive models, improving surgical outcomes, and managing patient risk within the field of cardiac procedures (Fig. 14).

**Figure 15.**
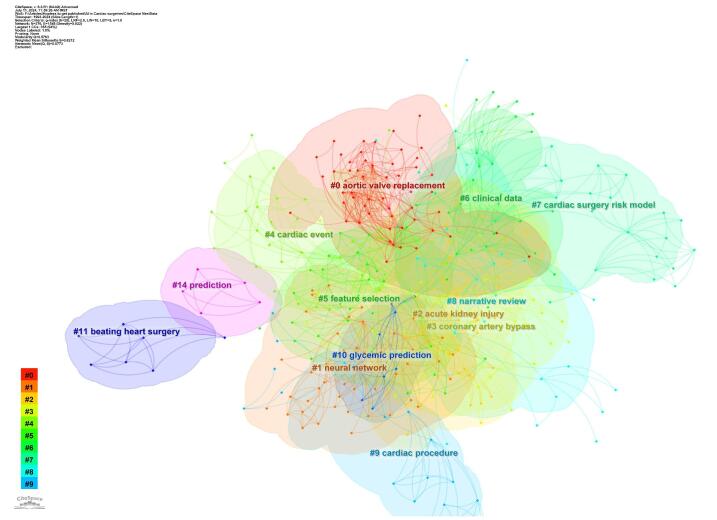
Cluster analysis of the topics in the field of artificial intelligence in cardiac procedures.

Through cluster analysis, 13 main clusters were identified, each representing significant areas of research focus. The key clusters included aortic valve replacement (#0), neural network cluster (#1), acute kidney injury cluster (#2), coronary artery bypass (#3), cardiac event (#4), feature selection (#5), clinical data (#6), cardiac surgery risk model cluster (#7), narrative review (#8), cardiac procedure (#9), glycemic prediction (#10), beating heart surgery (#11), and prediction (#14) (Fig. 15).

**Figure 16.**
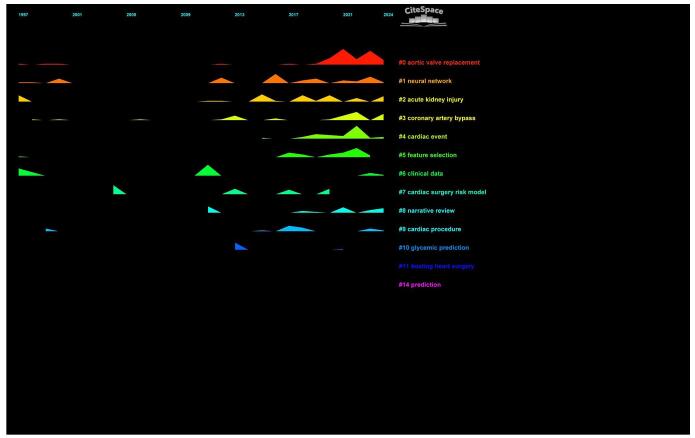
Time trend analysis of topics in the field of artificial intelligence in cardiac procedures.

The time trend analysis further illustrated the evolution of these clusters, showing how research priorities shifted over time. Newer clusters such as “aortic valve replacement,” “acute kidney injury,” and “coronary artery bypass” have gained more focus in recent years (Fig. 16).

## Discussion

The medical industry is fast changing due to AI, which is providing creative ways to enhance patient care, diagnosis, and treatment^[46]^. AI is being used in medicine in several areas, such as medical research, drug development, clinical decision support, medical imaging, and personalized medicine^[14,47-50]^. AI systems in medical imaging can analyze computed tomography scans, X-rays, magnetic resonance imaging (MRIs), and other images to find anomalies or lesions that human radiologists might overlook, improving diagnosis accuracy and speed^[51-53]^.

AI-powered clinical decision support tools assist healthcare providers by offering quick access to relevant information and research, helping them make more informed decisions about treatments and medications^[49]^. In drug development, AI accelerates the process by improving drug designs and identifying promising new drug combinations^[54]^. AI also enables more personalized healthcare approaches, such as customized virtual health assistance and precision medicine strategies tailored to individual patients^[55,56]^. Furthermore, AI is being used to enhance patient monitoring in critical care settings, predict disease outcomes, and streamline administrative tasks in healthcare facilities^[57,58]^. As AI continues to evolve, it has the potential to significantly improve healthcare efficiency, reduce costs, and ultimately lead to better patient^[12]^.

In this bibliometric study, utilizing data visualization and scientometrics analysis, we explored the current landscape of AI applications in cardiac procedures. We identified and presented the leading countries, their collaborative networks, and key institutions and authors in this domain. Additionally, we pinpointed the major research hot spots and uncovered emerging areas within the field.

The results of this bibliometric study showed an ongoing increase in the number of publications in the field. The growing volume of publications indicates a notable trend in both medical research and clinical practice. AI technologies are increasingly being incorporated into cardiology, improving diagnostic precision, predictive analytics, and tailored treatment strategies^[59,60]^. For example, algorithms for AI-driven image analysis can identify abnormalities in medical images with great accuracy, while predictive models sift through extensive patient data to uncover patterns and forecast cardiovascular diseases^[61-63]^.

The United States held the top position regarding the quantity of publications, the importance of contributions made to the discipline, and the existence of prominent institutions. Leading American institutions like Harvard Medical Center and the Mayo Clinic have engaged in a great deal of research and development, which is indicative of their dominance. From early risk prediction and diagnosis to postoperative risk assessment and management, these institutes have led the way in the development of numerous AI applications in cardiology^[64-68]^.

Among the most influential papers in the field, “Prediction of the Development of Acute Kidney Injury Following Cardiac Surgery by Machine Learning” stood out as the most cited^[31]^. Notably, 2 of the 10 most cited and 2 of the 10 most co-cited papers focused on using AI to predict acute kidney injury (AKI) in patients undergoing cardiac surgery. This emphasis highlights the potential of AI tools in forecasting AKI within this patient population^[69-73]^.

A key study by Tseng *et al*^[31]^, involving 671 cardiac surgery patients, demonstrated that 163 (24.3%) patients developed cardiac surgery-associated acute kidney injury (CSA-AKI) within the first postoperative week. Among the ML models evaluated, the random forest model achieved impressive performance with an area under curve (AUC) of 0.839. An ensemble model slightly surpassed this with an AUC of 0.843. The study concluded that ML models can effectively predict CSA-AKI, aiding in the optimization of postoperative treatment strategies and the reduction of complications following cardiac surgery.

The second most cited paper, “Machine Learning for Real-Time Prediction of Complications in Critical Care: A Retrospective Study”^[33]^, focused on using AI to predict complications in critical care settings. This study underscored the value of ML in real-time monitoring and decision support, facilitating timely and precise interventions to minimize complications during and after critical procedures. Additionally, the third most cited study, “Motion Estimation in Beating Heart Surgery”^[35]^, addressed the challenges posed by heart motion during minimally invasive surgery. The authors proposed algorithms for an advanced robotic surgery system that compensates for heart motion by tracking natural landmarks in the heart. This innovation highlights the role of ML in enhancing the precision and effectiveness of robotic-assisted cardiac surgeries.

Another interesting finding from our co-citation analysis revealed frequent co-citations between conventional risk models and contemporary AI studies. For instance, from the top 10 most co-cited studies, “EuroSCORE II” (2012)^[32]^ and “A Comparison of a Machine Learning Model with EuroSCORE II in Predicting Mortality after Elective Cardiac Surgery: A Decision Curve Analysis”^[34]^ were the most frequently cited. These studies provide a comparative analysis between traditional and AI-based predictive models. The frequent co-citation underscores the ongoing efforts to validate and potentially replace conventional risk models with more complex ML approaches, reflecting a significant shift towards embracing AI in clinical practice.

Our keyword analysis revealed that the most frequently occurring terms were “machine learning,” “mortality,” “cardiac surgery,” “artificial intelligence,” and “risk.” When examining the centrality of keywords within the network, the top five most central terms were “mortality,” “diagnosis,” “cardiac surgery,” and “artificial intelligence.” These keywords signify critical points of connection and influence within the research network. This trend highlights that the majority of articles focus on how AI can assist clinicians in reducing mortality rates and minimizing procedural risks.

AI has shown to have significant potential for improving cardiac procedure outcomes through better diagnostic accuracy, risk prediction, and treatment strategy optimization^[59,74-76]^. The results of recent studies demonstrate how AI can lower mortality after cardiac surgeries^[77,78]^.

According to a recent study, high-risk patients can be identified by AI-enabled electrocardiograms, which can result in prompt intensive care interventions and a considerable reduction in all-cause death^[79]^. It has been demonstrated that ML models are more accurate than conventional techniques at forecasting heart transplant outcomes, such as graft failure and mortality^[80]^. AI systems can also anticipate adverse outcomes and death in patients suffering from acute coronary syndrome, which makes early intervention and efficient treatment possible.

In terms of diagnostic and imaging capabilities, AI excels in analyzing cardiac imaging data, such as echocardiography and MRI, with high accuracy. This capability aids in the early detection of heart failure and other cardiac conditions. Additionally, AI-based devices for cardiac health monitoring, which utilize deep learning techniques to analyze heart sounds, can provide real-time, accurate diagnoses of various cardiac diseases^[81,82]^.

AI also plays a crucial role in optimizing treatment and management strategies. AI algorithms assist in clinical decision-making for valvular heart diseases by identifying risk factors and predicting surgical outcomes, thereby improving patient management^[75,83,84]^.

Support in resuscitation and emergency care is another area where AI shows promise^[85,86]^. AI can predict cardiac arrest and post-cardiac arrest outcomes, enhancing the effectiveness of resuscitation efforts and improving survival rates^[87,88]^.

In the cluster analysis, aortic valve replacement was identified as the most significant category. Additionally, transcatheter aortic valve implantation (TAVI) was noted as a new keyword in the time trend analysis. These findings suggest that the utilization of AI in valvular pathologies is being recognized as an emerging trend in the field. TAVI has been recognized for enhancing the management of severe aortic stenosis by providing less invasive alternatives to traditional surgical procedures^[89,90]^.

With advancements in AI and ML, predictive modeling has emerged as a critical tool for optimizing patient outcomes and procedural efficiency in TAVI. The prediction of post-TAVI outcomes, such as length of stay and complications, is regarded as crucial for the optimization of healthcare resource utilization and the enhancement of patient care. A random forest ML algorithm was employed by Judson *et al*^[91]^ to predict the length of stay following outpatient TAVI procedures, resulting in An AUC of 0.82 for short stays and 0.85 for long stays, outperforming traditional models. The significance of variables, including procedural duration and post-procedure physical therapy, in influencing recovery timelines was highlighted by their findings.

In a similar vein, a dataset of 92 363 TAVI cases was utilized by Khan *et al*^[92]^ to predict heart failure readmissions within 30 days post-procedure, achieving an AUC of 0.76 with their predictive model. This approach allows for the implementation of proactive management strategies aimed at mitigating heart failure complications, thereby enhancing the effectiveness of postoperative care.

Moreover, advanced ML models were developed by Abdul Ghaffar *et al* and Hernandez-Suarez *et al*^[93,94]^ that outperformed traditional scoring systems in the prediction of mortality outcomes post-TAVI.

## Limitation

This study has some limitations. Primarily relying on the Web of Science Core Collection, it may miss relevant publications. Publication bias could skew results, with positive findings more likely published. The analysis only includes data up to July 2024, potentially omitting recent studies. Varying study methodologies complicate comparisons. The focus on English-language publications and specific geographic regions may exclude important research. Lastly, the bibliometric design limits conclusions about AI’s practical impact on cardiovascular procedures, as it doesn’t incorporate clinical data.

## Conclusion

In conclusion, AI holds tremendous promise for transforming cardiac practice. Its applications span from improving diagnostic accuracy and risk prediction to optimizing treatment strategies and enhancing patient outcomes. AI-driven models can assist in early detection of complications, real-time monitoring, and precise interventions, ultimately reducing mortality and procedural risks. As evidenced by numerous studies, AI can outperform traditional methods in predicting outcomes such as AKI, heart transplant success, and postoperative recovery, thereby allowing for more informed and effective clinical decisions. Continued integration of AI in cardiac interventions will foster advancements in predictive analytics, personalized treatment plans, and overall procedural efficiency, leading to significant improvements in patient care and surgical success rates.

## Data Availability

Data can be provided on a reasonable request from corresponding author.

## Research registration unique identifying number (UIN)

The study is a bibliometric study and UIN is not applicable.
